# A Stochastic Delay Differential Model of Cerebral Autoregulation

**DOI:** 10.1371/journal.pone.0118456

**Published:** 2015-04-01

**Authors:** Simona Panunzi, Laura D’Orsi, Daniela Iacoviello, Andrea De Gaetano

**Affiliations:** 1 Consiglio Nazionale delle Ricerche, Istituto di Analisi dei Sistemi ed Informatica “Antonio Ruberti”, Rome, Italy; 2 Dipartimento di Ingegneria Informatica, Automatica e Gestionale “Antonio Ruberti”, Rome, Italy; Shenzhen Institutes of Advanced Technology, CHINA

## Abstract

Mathematical models of the cardiovascular system and of cerebral autoregulation (CAR) have been employed for several years in order to describe the time course of pressures and flows changes subsequent to postural changes. The assessment of the degree of efficiency of cerebral auto regulation has indeed importance in the prognosis of such conditions as cerebro-vascular accidents or Alzheimer. In the quest for a simple but realistic mathematical description of cardiovascular control, which may be fitted onto non-invasive experimental observations after postural changes, the present work proposes a first version of an empirical Stochastic Delay Differential Equations (SDDEs) model. The model consists of a total of four SDDEs and two ancillary algebraic equations, incorporates four distinct delayed controls from the brain onto different components of the circulation, and is able to accurately capture the time course of mean arterial pressure and cerebral blood flow velocity signals, reproducing observed auto-correlated error around the expected drift.

## Introduction

Autoregulation of blood flow denotes the intrinsic ability of an organ or a vascular bed to maintain a constant perfusion in the face of blood pressure changes [1]}. In particular, cerebral autoregulation (CAR) denotes the ability of the circulation to adapt to variations in hydrostatic pressures by means of compensating changes in heart rate, peripheral vascular resistances and venous capacitance, so as to maintain constant and adequate perfusion to the brain.

For the assessment and prognosis of the progression of some diseases, such as cerebrovascular accidents, Alzheimer and others [2–9], the evaluation of the adequacy of cerebral autoregulation provides important information. Compromised cerebral hemodynamics, such as reduced vasodilation, reaction to CO_2_ and other stimuli, may in fact, be related to reduced post-stenotic perfusion pressure.

Since the early 1990’s several scholars [10–19] have analyzed mathematically the vascular compensation mechanisms responsible for the regulation of cerebral blood flow.

A comprehensive, deterministic mechanistic model of the interplay of cerebral blood flow, cerebral blood volume, intracranial pressures and regulatory mechanisms, had been proposed by Ursino and Lodi [13]. This model, using measured arterial pressure (AP) as driving or input function, proved adequate to reproduce the observed cerebral blood flow velocity (CBFV) profile in human subjects undergoing transition from sitting- to—standing [20].

The Ursino-Lodi model was subsequently used [21] to analyze non-invasive measurements of cerebral blood flow velocity and arterial blood pressure on a healthy young subject undergoing postural changes. The Ursino-Lodi model was also used [21] to study variations in cerebral autoregulation between populations, even though the difficulty in estimating the many model parameters from non-invasive measurements of cerebral blood flow velocity and arterial blood pressure was underscored.

The first major limitation of this approach is that the time course of arterial pressure is simply taken as input function (thereby not explaining the determinants of the variations of arterial pressure itself) and so the time course of arterial pressure is not reproduced from hypotheses on the hemodynamic changes induced by the orthostatism. The second limitation is that the identification of the adequacy of cerebral autoregulation is difficult in a given single patient, because a mechanistic model requires many free parameters to be estimated from data and this limits the clinical applicability of the method. The third limitation is the inadequacy of the deterministic modeling of pressure and flow variables in capturing their irregular variations over time, produced by accidental muscle contractions, station adjustments, hormonal and neurological oscillations etc.

Following the same approach of Ursino and Lodi, Olufsen et al. [22] constructed a model that proposes to explain the morphology of both the arterial pressure signal and that of the corresponding cerebral blood flow velocity. The expected new (apparent) steady state reached after standing was correctly reproduced by this model. However, these authors pointed out that the predicted drop in cerebral blood flow velocity after standing was substantially smaller than observed. This shortcoming was mitigated by the same authors in a subsequent work, in which they detailed the construction of a comprehensive eleven-compartment model, based on a reconstruction of the flows and resistances in idealized, representative segments of the circulation. This improved model captured satisfactorily the behavior of the observed signals, even in the transient phase before establishment of a new equilibrium. The three most important model’s characteristics [18] are: the inclusion of non linear functions describing resistances of the large systemic arteries as functions of pressure; the inclusion of autonomic regulation; and the inclusion of an empirical model describing the dynamics of cerebral vascular resistance.

The plausible hypotheses for autonomic and cerebrovascular regulation on which the model is based, allow a good quantitative match with physiological observations, even if this match is obtained through a rather complex set of equations and parameters.

Chiu et al. [23] and Liau et al. [24] focused their studies on diabetic patients and applied time-domain cross-correlation as a technique to assess the relationship between blood pressure and cerebral blood flow velocity signals after postural changes. Liau et al. [25] extended the application of the same techniques to stroke patients and showed that mean arterial blood pressure changes in response to postural challenges were reduced in stroke patients.

The urgency of devising protocols to validate the reproducibility and ranges of the dynamic parameters extracted from these models, and the importance of developing multivariate models that take into account time-varying parameters was stressed by Panerai et al. [26].

Very recently a new empirical model has been presented in [27]. The model aims at representing the viscoelastic response of tissues (blood vessels, cerebral arteries…), which exhibit a continuous relaxation in response to stress and in the specific case to postural change from sitting to standing. The presented model is a mechanical analog model for predicting the CBFV in response to AP changes, incorporated in the model as an input function. While the strength of the model derives from its simplicity (it has only four parameters to be estimated), its major shortcoming lies in the lack of representing the physiological mechanisms intervening during the experiment.

With the above considerations in mind, a simpler model (if it could correctly reproduce observations) would clearly be of interest in the quest for practical assessment of the efficiency of CAR. At the same time, evident autocorrelated departures from the expected, smooth signal, indicate that stochastic elements are involved (stemming in all likelihood from moment-to-moment variations in muscle activity, hormonal concentrations, sympathetic/parasympathetic tone and other influences). Finally, it would therefore also be of interest to consider more prolonged variations, such as the likely continuing, slow increase in arterial pressure, after fast compensation, instead of assuming attainment of the Steady State within a few tens of seconds after standing.

The goal of the present work is therefore to describe a simple stochastic delay differential equations model of cardiovascular regulation during toilt-table or sitting-to-standing maneuvers. We aim to show that this model is able to reliably reproduce the time course of mean AP and CBFV time courses after changes in posture, inclusive of some auto-correlated oscillations around the expected signals.

## Materials and Methods

### Model description

In the present section a stochastic delay differential equations (SDDE’s) model for cerebral autoregulation is proposed. The model is composed of four compartments and each one of the four equations (1–4) represents one of the main components of overall cerebral regulation. The stochastic component of the model appears in Equation (1).

The model equations are as follows:

$$dC\left(t\right)=\left(k_{c}\left(C_{tgt}-C_{\Delta }^{\text{max}}\text{sin}\left(\rho \right)\right)-k_{xca}{\tilde{B}}_{1}\left(t\right)C\left(t\right)\right)dt+\sigma dW\left(t\right)$$ (1)

$$\frac{dA\left(t\right)}{dt}=k_{arch}H\left(t\right)C\left(t\right)R\left(t\right)-k_{xa}A\left(t\right)$$ (2)

$$\frac{dH\left(t\right)}{dt}=-k_{ha}{\tilde{B}}_{3}\left(t\right)H\left(t\right)+k_{h}$$ (3)

$$\frac{dF\left(t\right)}{dt}=k_{f}^{\text{max}}\frac{B{\left(t\right)}^{\nu }}{B_{50}^{\nu }+B{\left(t\right)}^{\nu }}-k_{xf}\tilde{F}\left(t\right)$$ (4)

with the initial conditions C(0) = C_0_, A(0) = A_0_, H(0) = H_0_, F(0) = F_0_. C (cm_H20_) is central venous pressure, A (mmHg) is arterial blood pressure, H (Hz) represents heart rate (as an index of sympathetic activity), and F (mL/sec) is cerebral blood flow velocity.

Moreover, two algebraically defined variables are introduced: the variable B (mmHg), representing brain arterial pressure, and variable R (mmHg/mL/sec), representing Peripheral Vascular Resistances as a function of brain arterial pressure $\tilde{B}$. R is modeled as a decreasing function of $\tilde{B}$ in order to represent the mechanism by which, if brain arterial pressure increases, peripheral arterioles dilate and peripheral resistance falls:

$$R\left(t\right)=R_{\text{inf}}+\left(R_{\text{max}}-R_{\text{inf}}\right)e^{-\lambda {\tilde{B}}_{2}}$$ (5)

Therefore, in the present formulation, Peripheral Resistance represents one of the control mechanisms of the cerebral autoregulation system, driven, with delay, by cerebral pressure.

Brain arterial perfusion pressure B differs from arterial blood pressure A by a pressure delta determined by the distance from the head to the heart (*dist*), multiplied by the *sine* of the tilt angle, that is the radians upright from the supine position:

B(t)=A(t)−a(ρ(t)) (6)

and

$$B(0)=B_{0}=A(0)-a(\rho (0))$$

with

a(ρ)=dist×sin(ρ(t))/1.36 (7)

where *ρ*(*t*) is the tilt angle at time *t*. In the present formulation we have set:

$$\rho \left(t\right)=\{\begin{array}{c} \rho_{0},{\text{t<t}}_{\text{0}}\\ \rho_{end}\text{, t}\geq {\text{t}}_{0} \end{array}$$ (8)

Notice that the model, as formulated, can be used to fit data from generic tilt-table maneuvers (with parameter ρ in eq. 1 taking values in [0, π/2], depending on the final tilt-angle) and also sitting-to-standing maneuvers (with the parameter ρ = π /2 or, which is the same, with sin(ρ) = 1, expressing a simple Heaviside step from one position to the other). In the following we will describe the (continuous) geometry relative to tilt-table experiments, since sitting-to standing experiments can be simply represented as theoretically instantaneous transitions between two positions, arbitrarily indexed by sin(ρ) = 0 and sin(ρ) = 1.

The delta pressure in cmH_2_O from the head to heart is converted to mmHg dividing by the conversion factor 1.36.

The first equation (Equation 1) describes the variation of central venous pressure (CVP, in the model indicated with C) over time. In order to allow the heart to pump sufficient blood to maintain cerebral perfusion pressure, and hence cerebral blood flow, it is supposed that compensating mechanisms act to maintain a sufficient level of CVP and ventricular filling. CVP is at steady state when the tendencies to increase CVP, necessary to maintain cerebral perfusion pressure, and to decrease CVP, when a sufficient level of brain perfusion pressure is obtained, equilibrate.

For *ρ* = 0 the subject lies supine and the ‘elastic’ CVP target value is *c*_*trgt*_, but when the subject undergoes a head-up tilt experiment, *ρ* increases proportionally to the tilt angle and the central venous pressure drops by a hydrostatic factor equal to $C_{\Delta }^{\text{max}}\text{sin}\left(\rho \right)$. After the maneuver, CVP increases again: the gain in CVP is due to both reduced loss and concurrent drop of suppressing (delayed) brain perfusion pressure (the multiplicative second term). After postural change the control mechanism restores CVP, allowing cardiac filling to be brought back to normal again. The system which drives CVP trend is subject to random fluctuations due to different mechanisms such as muscular contraction, respiration, deglutition and other uncontrolled factors, represented in the model by the random system noise σ*W*(*t*).

The second equation (Equation 2) describes variations of arterial blood pressure. These variations depend linearly on heart rate H, on CVP (these two multiplied together determine cardiac output) and on peripheral resistances R.

The third equation (Equation 3) describes the equilibrium between spontaneous increase and (delayed) brain perfusion pressure-dependent suppression of heart rate H. In fact, when the brain enjoys abundant perfusion, sympathetic tone may decrease, and heart rate may also correspondingly decreases [28].

The fourth equation (Equation 4) describes variations of cerebral blood flow velocity F as depending on current brain perfusion pressure B according to a Michaelis-Menten relationship: cerebral flow increases with increasing B, reaching a maximum level $k_{f}^{\text{max}}$ (in order to represent self-protecting cerebral vasoconstriction mechanisms, which may be missing in some pathological conditions). The parameter *B*_50_ represents instead the brain perfusion pressure level at which a half-maximal rate of flow increase is obtained. The spontaneous decay of the flow is represented by the second term on the right hand of the equation, where the variable F is delayed, $\tilde{F}$.

All the delayed variables in the model appear with a tilde hat on the top of the variable. In the present model all the considered delays are distributed: the influence of the variable on the dynamics of the system at a certain time *t* is based on previous values assumed by the variable until time *t*, weighted with a delay kernel function according to the following definition (Y indicates a generic variable):

$$\tilde{Y}=\int_{0}^{\infty }\omega (s)Y(t-s)ds$$ (9)

with $\omega \left(s\right)=\alpha^{0}se^{-\alpha s}\text{ and }\int_{0}^{\infty }\omega (s)ds=1$

The exponential delay kernel *ω(s*) is parameterized by the parameter α, which is greater than zero: for α large, recent values carry more weight. For each of the delayed variables considered in the model (${\tilde{B}}_{1}\left(t\right)$,${\tilde{B}}_{2}\left(t\right)$,${\tilde{B}}_{3}\left(t\right)$,$\tilde{F}\left(t\right)$) a different parameter α of the kernel function is considered.

**Fig 1:**
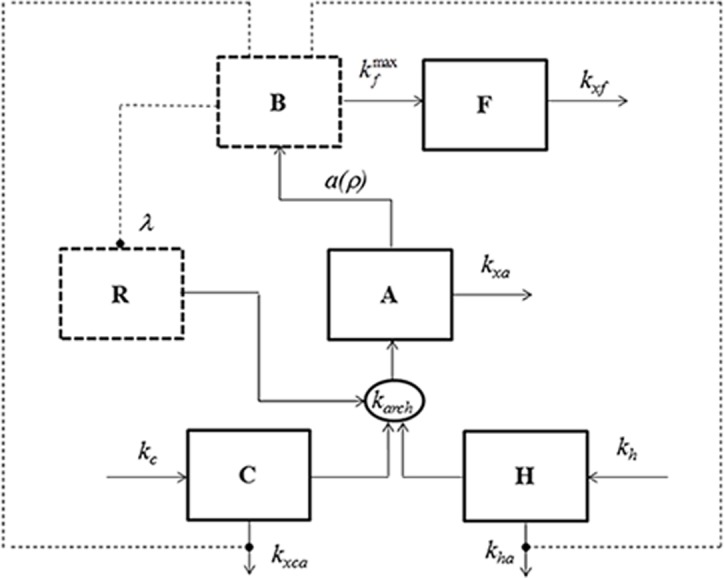
Block diagram of the ESDDeCAR-02 model. Blocks in continuous lines represent the model state variables; blocks in dashed lines represent the two defined algebraic variables.

Fig. 1 shows the schematic representation of the model.

The system admits a continuum of different equilibria, indexed by *ρ*, when the model drift (that is the deterministic component only) is considered. Each of the equations have two steady state conditions, before the maneuver and at infinite time after its completion:

$$A_{0}=R_{0}C_{0}H_{0}k_{arch}/k_{xa}$$ (10)

$$A_{end}=\left(k_{arch}H_{end}C_{end}R_{end}\right)/k_{xa}$$ (11)

$$B_{0}=A_{0}-a(\rho (t_{0}))=A_{0}-\frac{dist}{1.36}sin(0)=A_{0}$$ (12)

$$B_{end}=A_{end}-a(\rho (t_{end}))=A_{end}-\frac{dist}{1.36}sin(\rho (t_{end}))=A_{end}-\frac{dist}{1.36}$$ (13)

$$H_{end}=\left(A_{0}H_{0}\right)/\left(A_{end}-(\frac{dist}{1.36})sin(\rho_{end})\right)$$ (14)

$$F_{0}=\frac{k_{f}^{max}}{k_{xf}}\frac{B_{0}^{\nu }}{B_{50}^{\nu }+B_{0}^{\nu }}$$ (15)

$$F_{end}=F_{0}\frac{(B_{50}^{\nu }+B_{0}^{\nu })B_{end}}{(B_{50}^{\nu }+B_{end}^{\nu })B_{0}^{\nu }}$$ (16)

$$R_{end}=R_{inf}+(R_{max}-R_{inf})e^{-\lambda (A_{end}-(\frac{dist}{1.36})sin(\rho_{end}))}$$ (17)

While *H*_0_ and *R*_0_ are supposed to be assigned (fixed).

From the steady state conditions the following parameters are also determined:

$$k_{ha}=\frac{k_{h}}{A_{0}H_{0}}$$ (18)

$$C_{tgt}=\frac{k_{xca}B_{0}C_{0}}{k_{c}}$$ (19)

$$C_{\Delta }^{max}=C_{tgt}-\frac{k_{xca}}{k_{c}}B_{end}C_{end}$$ (20)

$$R_{max}{\text{= (k}}_{xa}A_{0}e^{\lambda A_{0}}\text{)}/(k_{arch}H_{0}C_{0})-e^{\lambda A_{0}}R_{inf}(1-e^{-\lambda A_{0}})$$ (21)

Table 1 reports the model parameters along with their description, units of measurement and values used in the simulations.

**Table 1:** List and description of parameters values used in the simulation.

Parameter	Unit of Measurements	Description	Value
*k*_*c*_	/sec	Rate of central venous pressure increase	1
*C*_*tgt*_	cm_H20_	Central venous pressure target value	determined
$C_{\Delta }^{max}$	cm_H20_	Proportional constant drop of central venous pressure target following the tilt experiment	determined
*k*_*xca*_	/sec/mmHg	Central venous pressure decrease rate per mmHg of brain arterial pressure	0.01
ρ	radians	tilt angle	0,π/2
*dist*	cm	Distance between head and heart	30
*C*_*0*_	cm_H20_	Central venous pressure value before the tilt experiment	8
*C*_*end*_	cm_H20_	Central venous pressure value at equilibrium after the tilt experiment	4
*k*_*arch*_	mL/cm_H2O_/bpm	Third-order arterial pressure increase rate	0.2
*k*_*xa*_	/sec	Arterial pressure decrease rate	0.5
*A*_*0*_	mmHg	Arterial pressure value before the tilt experiment	determined
*A*_*end*_	mmHg	Arterial pressure value at equilibrium after the tilt experiment	determined
*k*_*ha*_	/sec/mmHg	Heart rate decrease rate per mmHg of brain arterial pressure	determined
*k*_*h*_	bpm/sec	Spontaneous increase of heart rate	0.05
*H*_*0*_	bpm	Heart rate value before the tilt experiment	60
*H*_*end*_	bpm	Heart rate value at equilibrium after the tilt experiment	determined
$k_{f}^{max}$	mL/sec/sec	Maximal increase in cerebral blood flow velocity	28.9
*k*_*xf*_	/sec	Cerebral blood flow velocity decrease rate	0.5
*F*_*0*_	mL/sec	Cerebral blood flow velocity value before the tilt experiment	determined
*F*_*end*_	mL/sec	Cerebral blood flow velocity value at equilibrium after the tilt experiment	determined
*ν*	#	Rapidity with which cerebral blood flow velocity increase reaches its maximum with increasing B	1
*B*_*50*_	mmHg	Brain arterial pressure level at which an half-maximal increment of cerebral blood flow velocity is obtained	40
*B*_*0*_	mmHg	Brain arterial pressure value before the tilt experiment	determined
*B*_*end*_	mmHg	Brain arterial pressure value at equilibrium after the tilt experiment	determined
*R*_*inf*_	mmHg/mL/sec	Minimum value obtained for peripheral vascular resistance	0
*R*_*max*_	mmHg/mL/sec	Maximum value obtained for peripheral vascular resistance	determined
λ	/mmHg	Rate of decay of peripheral vascular resistance with increasing brain arterial pressure	0.035
*R*_*0*_	mmHg/mL/sec	Peripheral vascular resistance value before the tilt experiment	0.47
*R*_*end*_	mmHg/mL/sec	Peripheral vascular resistance value at equilibrium after the tilt experiment	determined
*α*_*i*_	/sec	Delay kernel rate constant for delay kernel *i*	0.01,0.1,0.2,0.6
*t*_*0*_	sec	Time of tilt maneuver	30
σ	cm_H20_/sec^1/2^	CVP volatility	0.5

The model was implemented in Matlab using a fixed-step, fourth-order Runge-Kutta numerical integration scheme [29].

### Qualitative analysis of the model solutions

The present deterministic qualitative analysis refers to the drift part of model (1–8) only (without the additional stochastic term *σdW* in (Eq. 1)).

The following subsections investigate the conditions for which the solutions are positive and the model is persistent, report the proof that a unique positive equilibrium point exists and provide asymptotic local stability analysis.

#### Positivity of the model solutions and model persistence

**Theorem 1.** *The system (1)*, *(2)*, *(3) and (4) admits positive solutions for any positive initial condition*.

***Proof*.** Let *C*(0)>0. According to the continuity of the solution of a differential equation, *C*(*t*) would become non-positive if there existed a t*>0 such that *C*(t*) = 0, *C*(*t*)>0 for any 0 ≤*t* <t*, and ${\frac{dC}{dt}|}_{t=t^{*}}\leq 0$, which cannot be, because under these hypotheses:

$$\begin{array}{l} {\frac{dC}{dt}|}_{t=t^{*}}=k_{c}(C_{tgt}-C_{\Delta }^{\max}\sin(\rho ))-k_{xca}{\tilde{B}}_{1}(t^{*})C(t^{*})=k_{c}(C_{tgt}-C_{\Delta }^{\max}\sin(\rho ))=\\ \{\begin{array}{c} k_{c}C_{tgt}\text{>0 if}t^{*}<t^{0}\\ k_{c}(C_{tgt}-C_{\Delta }^{\max})=\frac{k_{xca}}{k_{c}}B_{end}C_{end}\text{>0 if}t^{*}\geq t^{0} \end{array} \end{array}$$ (22)

This proves that *C*(*t*)>0 (it never vanishes and it is always positive). Similarly it can be proven that, if *H*(0)>0, also *H*(*t*) never vanishes and is always positive: let *H*(0)>0 and assume that ∃t*>0 such that *H*(t*) = 0 and *H(t*)>0 for any 0≤ t <t*. Then it cannot be that ${\frac{dH}{dt}|}_{t=t^{*}}\leq 0$, because:

$${\frac{dH}{dt}|}_{t=t^{*}}=-k_{ha}{\tilde{B}}_{3}(t^{*})H(t^{*})+k_{h}=k_{h}>0.$$ (23)

For (equation 2) let *A*(0)>0. *A*(*t*) would become non-positive if there existed a t*>0 such that *A*(t*)>0, for any 0 ≤ t<t*, and ${\frac{dA}{dt}|}_{t=t^{*}}\leq 0$, which cannot be, because in this case:

$${\frac{dA}{dt}|}_{t=t^{*}}=k_{arch}H(t^{*})C(t^{*})R(t^{*})-k_{xa}A(t^{*})=k_{arch}H(t^{*})C(t^{*})R(t^{*})>0.$$ (24)

This proves that *A*(*t*) > 0.

For (equation 3) let *F*(0)>0. Again,*F*(*t*) would become non-positive if there existed a t*>0 such that *F*(t*) = 0, *F*(*t*)>0 for any 0 ≤ *t* <t*, and ${\frac{dF}{dt}|}_{t=t^{*}}\leq 0$.

$$\frac{dF}{dt}{|}_{t=t^{*}}=k_{f}^{\max}\frac{B{\left(t^{*}\right)}^{\nu }}{B_{50}^{\nu }+B{\left(t^{*}\right)}^{\nu }}-k_{xf}\tilde{F}(t^{*})=\{\begin{array}{c} k_{f}^{\max}\frac{A{\left(t^{*}\right)}^{\nu }}{B_{50}^{\nu }+A{\left(t^{*}\right)}^{\nu }}-k_{xf}\tilde{F}(t^{*})>0\;\;\;\;\;\;\;\text{if}t^{*}<t_{0}\\ k_{f}^{\max}\frac{{\left(A(t^{*})-dist/1.36\right)}^{\nu }}{B_{50}^{\nu }+{\left(A(t^{*})-dist/1.36\right)}^{\nu }}-k_{xf}\tilde{F}(t^{*})>0\;\;\text{if}t^{*}\geq t_{0} \end{array}$$ (25)

At equilibrium, $\tilde{F}(t^{*})=F(t^{*})=0$, from which it follows that dFdt|t=t*={kfmaxA(t*)νB50ν+A(t*)ν   ift*<t0kfmax(A(t*)-dist/1.36)νB50ν+(A(t*)-dist/1.36)ν  ift*≥t0, since *A*(t*)–*dist*/1.36 > 0 due to physiological considerations (A(t) is larger than 29 mmHg in all humans still alive). This means that at equilibrium *F*(*t*) is always strictly positive.

In the transient, the behavior of *F*(*t*) depends on $\tilde{F}(t^{*})$. Since the analytic investigation of the conditions, for which the positivity of *F*(*t*) always holds, is very cumbersome, a numerical exploration of the behavior of *F*(*t*), as depending on a range of values of the parameters *α*_*F*_ and *R*_o_, has been performed.

**Fig 2:**
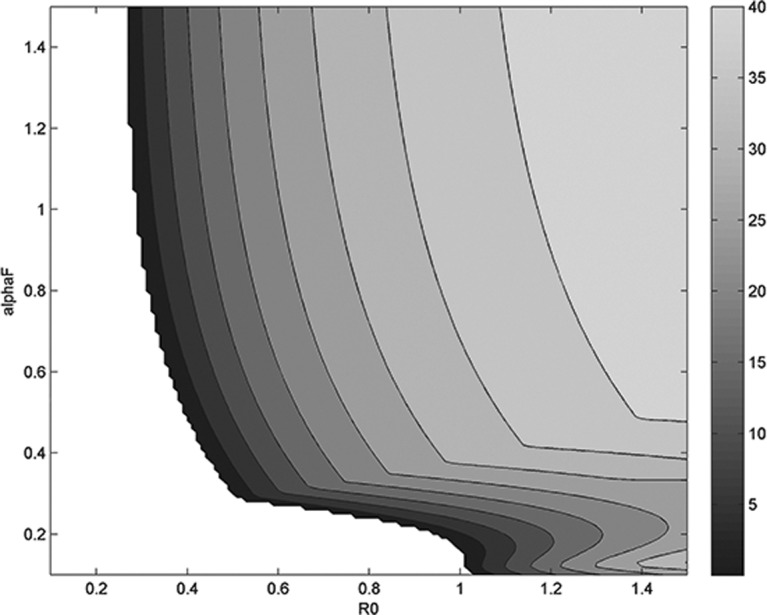
Behavior of the minimum of the cerebral blood flow velocity (CBFV) trend over time varying parameters *α*_*F*_ and *R*_0_. White region represents negative (unacceptable) values of the minimum, graded colored region represents the (positive) value of the minimum of the CBFV over the perturbation time interval.

Fig. 2 show the behavior of the minimum of the cerebral blood flow velocity time course over the duration of the experiment. A region of the plain is explored, determined by a range of values of *α*_*F*_ from 0.1 to 1.5 and by a range of *R*_0_ from 0.1 to 1.5 (mmHg/mL/sec). The color coding shows with darker color smaller but positive values of the *min(F)*, while white denotes negative values of CBFV, which are of course physiologically unacceptable.

**Theorem 2**. *The system of equations (1–4) is persistent.*

***Proof*.** Recall that a model is persistent if there exists a pair of positive real numbers (m, M) such that:

$$\exists \;\overset{-}{t}:\;0<m<X_{i}(t)<M<+\infty ,\;\forall t\geq \overset{-}{t},$$ (26)

for each component *X*_*i*_ of the state vector. Denote:

$$\begin{array}{cc} C_{m}=lim{inf}_{t\to +\infty }C(t), & C_{M}=lim{sup}_{t\to +\infty }C(t),\\ A_{m}=lim{inf}_{t\to +\infty }A(t), & A_{M}=lim{sup}_{t\to +\infty }A(t),\\ H_{m}=lim{inf}_{t\to +\infty }H(t), & H_{M}=lim{sup}_{t\to +\infty }H(t),\\ F_{m}=lim{inf}_{t\to +\infty }F(t), & F_{M}=lim{sup}_{t\to +\infty }F(t). \end{array}$$

The proof is achieved by proving the following eight statements:

$$\begin{array}{cc} C_{M}<+\infty , & C_{m}>0,\\ A_{M}<+\infty , & A_{m}>0,\\ H_{M}<+\infty , & H_{m}>0,\\ F_{M}<+\infty , & F_{m}>0. \end{array}$$

*Step 1*. In order to show the boundedness of the evolution of central venous pressure, assume that *C*_*M*_ = +∞

Since *C* is by definition differentiable and since *C*_*M*_ = limsup_t→+∞,_ we can define a sequence of time points {*t*_*n*_} such that:

- *t*_*n*_ *→*+∞ as *n→*+∞
- *C*(*t*_*n*_)*→*+∞ as *n→*+∞
- $${\frac{dC(t)}{dt}|}_{t=t_{n}}>0\;\forall t_{n}$$ (27)

If we consider (equation 1) at each of time points *t*_*n*_, we find that ${\frac{dC}{dt}|}_{t=t_{n}}=k_{c}(C_{tgt}-C_{\Delta }^{max}sin(\rho ))-k_{xca}B_{1}(t_{n})C(t_{n})\to -\infty$ which is a contradiction, so that *C*_*M*_ < + ∞.

*Step 2*. In order to show the boundedness of the evolution of arterial blood pressure, heart rate, cerebral blood flow velocity, assume that *A*_*M*_ = *H*_*M*_ = *F*_*M*_ *= + ∞*. From the same considerations as in (27) it should be:

*A*(*t*_*n*_)→ + ∞,*H*(*t*_*n*_)→ + ∞,*F*(*t*_*n*_)→ + ∞ and ${\frac{dA}{dt}|}_{t=t_{n}}>0,\;{\frac{dH}{dt}|}_{t=t_{n}}>0,\;{\frac{dF}{dt}|}_{t=t_{n}}>0\;\forall {\text{t}}_{n}$.

However:

$${\frac{dA}{dt}|}_{t=t_{n}}=k_{arch}H(t_{n})C(t_{n})R(t_{n})-k_{xa}A(t_{n})\to -\infty ,$$

$${\frac{dH}{dt}|}_{t=t_{n}}=-k_{ha}{\tilde{B}}_{3}(t_{n})H(t_{n})+k_{h}\to -\infty ,$$

$${\frac{dF}{dt}|}_{t=t_{n}}=k_{f}^{\max}\frac{B{\left(t_{n}\right)}^{\nu }}{B_{50}^{\nu }+B{\left(t_{n}\right)}^{\nu }}-k_{xf}\tilde{F}(t_{n})\to -\infty ,$$

which are contradictions, so that *A*_*M*_ < +∞, H_*M*_ < + ∞,F_*M*_ < + ∞.

*Step 3*. From Step 1, it follows that *C*_*m*_ ≤ *C*_*M*_ < + ∞. Since *C* is by definition differentiable and since *C*_*m*_ liminf_*t→+∞*_ *C*(*t)*, we can define a sequence of time points {*t*_*n*_} such that:

- *t*_*n*_→ +∞ as *n*→ +∞
- *C*(*t*_*n*_)→*C*_*m*_ as *n*→ +∞
- ${\underset{n\to +\infty }{lim}\frac{dC(t)}{dt}|}_{t=t_{n}}\text{=0 }\forall t_{n}$

which means:

0=limn→∞[kc(Ctgt-CΔmaxsin(ρ))-kxcaB(tn)C(tn)]≥(kc(Ctgt-CΔmaxsin(ρ))-kxca(AM-a(ρ))Cm(t))⇒kxca(AM-a(ρ))Cm≥kc(Ctgt-CΔmaxsin(ρ)) (28)

According to inequality (28), *Cm*>0 because $C_{m}>\frac{k_{c}(C_{tgt}-C_{\Delta }^{max}sin(\rho ))}{k_{xca}(A_{M}-a(\rho ))}=B_{end}C_{end}/\left(A_{M}-a(\rho )\right)>0$

*Step 4*. From Step 2, it follows that *H*_*m*_ ≤ *H*_*M*_ <+∞, *A*_*m*_≤ *A*_*M*_ <+∞ and. *F*_*m*_≤ *F*_*M*_ <+∞ From the same considerations as in step 3 it follows that, for each state variable H, A and F, it is possible to define a sequence of time points {*t*_*nH*_}{*t*_*nA*_} and {*t*_*nF*_} such that:

tnH→+∞ as nH→+∞H(tnH)→Hm as nH→+∞tnA→+∞asnA→+∞A(tnA)→AmasnA→+∞tnF→+∞asnF→+∞F(tnF)→FmasnF→+∞.

and

limn→+∞dH(t)dt|t=tnH=0,∀tnH limn→+∞dA(t)dt|t=tnA=0, ∀tnAlimn→+∞dF(t)dt|t=tnF=0, ∀tnF 

which means:

$$\begin{array}{l} 0=\underset{n_{H}\to \infty }{\lim}\left(-k_{ha}B_{3}(t_{n_{H}})H(t_{n_{H}})+k_{h}\right)\geq -k_{ha}(A_{M}-a(\rho ))H_{m}+k_{h}\Rightarrow H_{m}\geq k_{h}/k_{ha}(A_{M}-a(\rho ))>0,\\ 0=\underset{n_{A}\to \infty }{\lim}(k_{arch}H(t_{n_{A}})C(t_{n_{A}})R(t_{n_{A}})-k_{xa}A(t))\geq k_{arch}H_{m}C_{m}R(t_{n})-k_{xa}A_{m}\Rightarrow A_{m}\geq \frac{k_{arch}H_{m}C_{m}R(t_{n})}{k_{xa}}>0,]\\ 0=\underset{n_{F}\to \infty }{\lim}\left(k_{f}^{\max}\frac{B{\left(t_{n_{F}}\right)}^{\nu }}{B_{50}^{\nu }+B{\left(t_{n_{F}}\right)}^{\nu }}-k_{xf}F(t_{n_{F}})\right)\geq k_{f}^{\max}\frac{{\left(A_{m}-a(\rho )\right)}^{\nu }}{B_{50}^{\nu }+{\left(A_{m}-a(\rho )\right)}^{\nu }}-k_{xf}F_{m}\Rightarrow F_{m}\geq \frac{k_{f}^{\max}}{k_{xf}}\frac{{\left(A_{m}-a(\rho )\right)}^{\nu }}{B_{50}^{\nu }+{\left(A_{m}-a(\rho )\right)}^{\nu }}. \end{array}$$

#### Existence of positive equilibrium points

It is shown here that each equation of the model (1–4) admits positive equilibrium points over a set indexed by *ρ*.

**Theorem 3**. *System (1–4) has a unique positive equilibrium point for each ρ*.

As derived above, for *t* = 0 (*a*(*ρ*(*t*)) = 0 the equilibrium point is given by (*C*_0_,*A*_0_,*H*_0_,*F*_0_), and for *t* = *t*_*end*_ (*a*(*ρ*_*end*_) = 1) the equilibrium point is given by (*C*_*end*_,*A*_*end*_,*H*_*end*_,*F*_*end*_).

For *t*≠0 and *t*≠*t*_*end*_ the equilibrium points of (1–4) satisfy the following algebraic equations:

$$k_{c}(C_{tgt}-C_{\Delta }^{max}sin(\rho ))-k_{xca}B_{e}C_{e}=0$$ (29)

$$k_{arch}H_{e}C_{e}R_{e}-k_{xa}A_{e}=0$$ (30)

$$-k_{ha}B_{e}H_{e}+k_{h}=0$$ (31)

$$k_{f}^{max}\frac{B_{e}{}^{\nu }}{B_{50}^{\nu }+B_{e}{}^{\nu }}-k_{xf}F_{e}=0$$ (32)

from which it follows that:

$$C_{e}=\frac{k_{c}(C_{tgt}-C_{\Delta }^{max}sin(\rho ))}{k_{xca}(A_{e}-a(\rho ))}$$ (33)

$$H_{e}=\frac{k_{h}}{k_{ha}(A_{e}-a(\rho ))}$$ (34)

$$F_{e}=\frac{k_{f}^{max}{\left(A_{e}-a(\rho )\right)}^{\nu }}{k_{xf}{\left(B_{50}^{\nu }(A_{e}-a(\rho ))\right)}^{\nu }}$$ (35)

$$A_{e}=\frac{k_{arch}}{k_{xa}}H_{e}C_{e}R_{e}$$ (36)

$$B_{e}=A_{e}-a(\rho )$$ (37)

$$R_{e}=R_{inf}+(R_{max}-R_{inf})e^{-\lambda (A_{e}-a(\rho ))}$$ (38)

Substituting (33–35) and (37–38) in (36):

$$\varphi (A_{e})=-A_{e}^{3}+2A_{e}^{2}a(\rho )-Aa{\left(\rho \right)}^{2}+\frac{k_{arch}k_{h}k_{c}(C_{tgt}-C_{\Delta }^{max}sin(\rho ))}{k_{ha}k_{xca}k_{xa}}((R_{max}-R_{inf})e^{-\lambda (A_{e}-a(\rho ))}+R_{inf})=0.$$

Notice that

$$\varphi (0)=\frac{k_{arch}k_{h}k_{c}(C_{tgt}-C_{\Delta }^{max}sin(\rho ))}{k_{ha}k_{xca}k_{xa}}\times ((R_{max}-R_{inf})e^{-\lambda (A_{e}-a(\rho ))}+R_{inf})>0$$

and

dφdA=-3A2+4Aa(ρ)-a(ρ+-λkarchkhkc(Ctgt-CΔmaxsin(ρ))khakxcakxa×(Rmax-Rinf)e-λ(A-a(ρ))<0,∀A>43a(ρ).

This condition is satisfied ∀*ρ*(*t*), in fact

$a_{max}=a(\rho_{max}(t))=dist\times sin(\frac{\pi }{2})/1.36\approx 22\Rightarrow \frac{4}{3}a_{max}\approx 29$ and due again to physiological considerations it is always *A*≫29mmHg.

From the above it follows that *φ* (·) is a decreasing function for positive argument, starting from a positive value at zero, and hence that it has at most one positive root. The existence of such a root is ensured by the limit:

$$\underset{A\to \infty }{lim}\varphi (A)=\underset{a\to \infty }{lim}((-A^{3}+4A^{2}a-Aa^{2})+\frac{k_{arch}k_{h}k_{c}(C_{tgt}-C_{\Delta }^{max}sin(\rho ))}{k_{ha}k_{xca}k_{xa}}((R_{max}-R_{inf})e^{-\lambda (A-a(\rho ))}+R_{inf}))=-\infty .$$

#### Asymptotic local stability analysis

In order to study the local stability of the equilibrium points (indexed by *ρ*), the system (1)-(4) is linearized around its generic equilibrium point (*C*_*e*,_ *A*_*e*,_ *H*_*e*,_ *F*_*e*_)

Adopting a change of variable, the delayed variable $\tilde{F}$can be written as:

$$\tilde{F}=\alpha_{F}^{2}\int_{0}^{+\infty }sF(t-s)e^{-\alpha_{F}s}ds=\;\alpha_{F}^{2}\int_{-\infty }^{t}(t-\theta )F(\theta )e^{-\alpha_{F}(t-\theta )}d\theta .$$

Denoting:

x1(t)=C(t)x2(t)=A(t)x3(t)=H(t)x4(t)=F(t)x5(t)=∫-∞t(t-θ)F(θ)e-αF(t-θ)dθx6(t)=∫-∞tF(θ)e-αF(t-θ)dθ

The following 6-dimentional ordinary differential system is obtained:

$$\begin{array}{l} {\dot{x}}_{1}=\frac{dC}{dt}=k_{c}(C_{tgt}-C_{\Delta }^{\max}sen(\rho ))-k_{xca}{\tilde{B}}_{1}x_{1}\\ {\dot{x}}_{2}=\frac{dA}{dt}=k_{arch}x_{1}x_{3}R-k_{xa}x_{2}\\ {\dot{x}}_{3}=\frac{dH}{dt}=-k_{ha}{\tilde{B}}_{3}x_{3}+k_{h}\\ {\dot{x}}_{4}=\frac{dF}{dt}=k_{f}^{\max}\frac{B^{\nu }}{B_{50}^{\nu }+B^{\nu }}-k_{xf}\alpha_{F}^{2}x_{5}\\ {\dot{x}}_{5}=-\alpha_{F}x_{5}+x_{6}\\ {\dot{x}}_{6}=x_{4}-\alpha_{F}x_{6} \end{array}$$ (39)

Taking into account the extended system (39), the local asymptotic stability of the equilibrium point

$$X_{eq}={\left(\begin{array}{cccccc} C_{eq} & A_{eq} & H_{eq} & F_{eq} & \frac{F_{eq}}{\alpha_{F}^{2}} & \frac{F_{eq}}{\alpha_{F}} \end{array}\right)}^{T}$$

is achieved if and only if all the roots of the characteristic polynomial associated with the Jacobian

$$J_{eq}=\left[\begin{array}{cccccc} a & 0 & 0 & 0 & 0 & 0\\ b & c & d & 0 & 0 & 0\\ 0 & 0 & e & 0 & 0 & 0\\ 0 & 0 & 0 & 0 & f & 0\\ 0 & 0 & 0 & 0 & g & 1\\ 0 & 0 & 0 & 1 & 0 & h \end{array}\right]$$ (40)

computed in *X*_*eq*_ have negative real part. The elements in (40) are defined as follows:

a=J11=-kxcaBeq=-kxca(Aeq-a(ρeq))b=J21=karchx3eqReqc=J22=-kxad=J23=karchx1eqReqe=J33=-khaBeqf=J45=-kxfαF2g=J55=-αFh=J66=-αF

After computation, the characteristic polynomial is:

$$p(\lambda )=det(J_{eq}-\lambda I)=[-\prod_{i=1}^{3}\left(p_{i}-\lambda \right)](\lambda^{3}-(g+h)\lambda^{2}+gh\lambda -f)$$ (41)

where *p*_1_ = *a*, *p*_*2*_ = *c*, *p*_*3*_ = *e*. The polynomial (41) is zero if and only if (*p*_*i*_ – λ) = 0∀*i* and (λ^3^ -(g+h)λ^2^ + gh λ-*f*) = 0.

It follows that $(p_{i}-\lambda )=0\;\forall i\Leftrightarrow \lambda =p_{i}\;\forall i$, where *a*, *c* and *e* are negative values.

For the study of the sign of the eigenvalues associated with the polynomial (λ^3^ -(g+h)λ^2^ + gh λ-*f*) the Routh-Hurwitz criterion is used. The first column of the Routh-Hurwitz table is:

$${\left[\begin{array}{cccc} 1 & -2g & \frac{2g^{3}-f}{2g} & -f \end{array}\right]}^{T},$$ (42)

and we have to determine for which conditions on parameters *g* and *f* the elements of (42) assume positive values. These conditions assure that the roots of the polynomial all have negative real part:

*-2g* is always positive, since g<0.
- $\frac{2g^{3}-f}{2g}>0\Leftrightarrow 2g^{3}-f<0$ which is true if $2g^{3}+k_{xf}g^{2}<0\Leftrightarrow g<\frac{-k_{xf}}{2}\Leftrightarrow \alpha_{F}>\frac{k_{xf}}{2}$
-*f* is always positive, since *f<0*.

The condition in ii) translates into the standard requirement for delayed systems that, in order to guaranteed model stability, a sufficiently small (average) delay on the considered delayed state variable *F* is necessary: in fact, the larger the parameter *α*_*F*_, the smaller the associated average delay.

## Results

**Fig 3:**
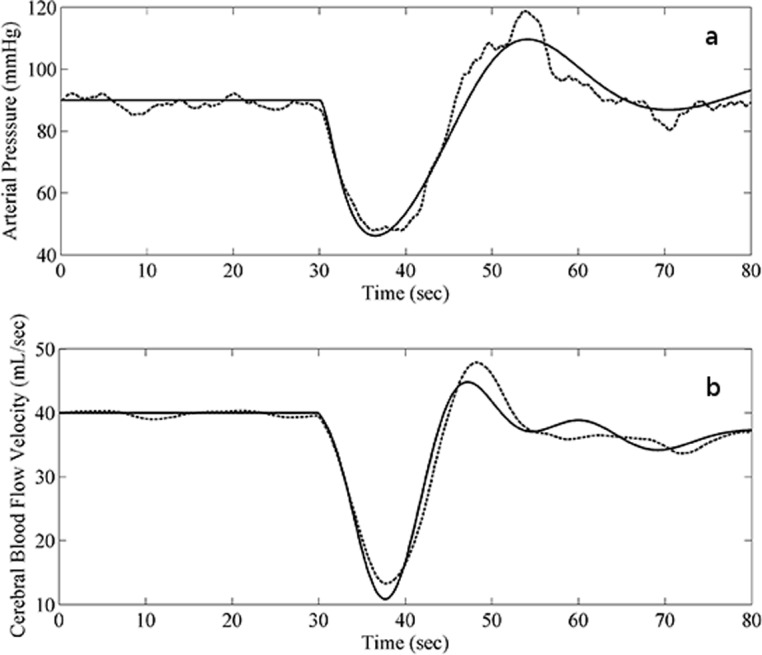
Simulated model behavior in normal physiological conditions. Panel A: time course of model-simulated arterial pressure (mmHg) (dashed line: stochastic model, continuous line: model drift). Pressure drops of about 40 mmHg after standing, with a nadir at nearly 5–8 seconds post-maneuver, climbs back within another 10 to 15 seconds with an overshoot of approx 20 mmHg before stabilizing. Panel B: time course of model-simulated cerebral blood flow velocity (CBFV,mL/sec) (dashed line: stochastic model, continuous line: model drift). CBFV drops of about 30 mL/sec after standing, with a nadir at 5 to 8 seconds post-maneuver, climbs back within another 10–15 seconds with an overshoot of approx 5 mL/sec before stabilizing.

**Fig 4:**
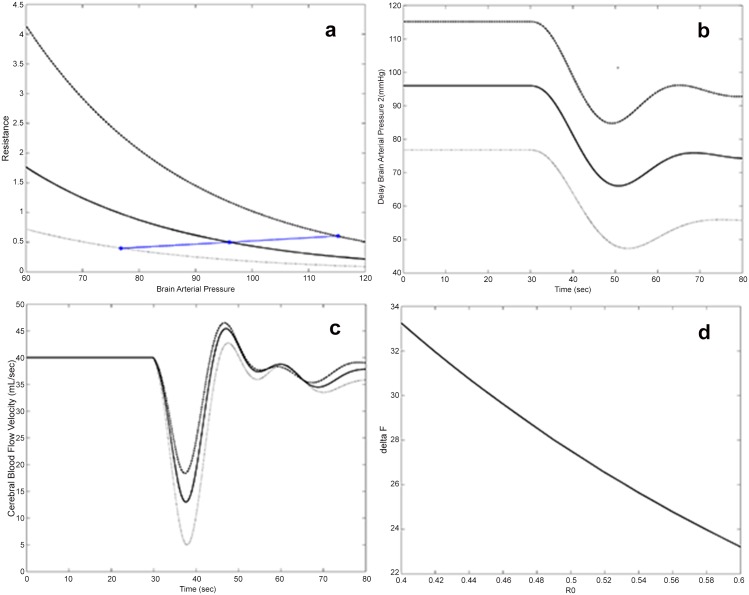
Simulated model behavior in abnormal physiological conditions obtained by varying *R*_0_. Panel A reports model-simulated Resistance (R, mmHg/mL/sec) as an algebraic function of brain arterial pressure (mmHg) for different values of *R*_*0*_. Moreover for each value of *R*_*0*_ the equilibrium points of brain arterial pressure (and corresponding resistance) are shown. Panel B and C report the time course of model-simulated delayed brain arterial pressure (mmHg) and of cerebral blood flow velocity (mL/sec) respectively, in correspondence of the different values of *R*_0_. Panel D reports delta CBFV (difference between the lowest value of CBFV over time and *F*_0_) as a function of *R*_0_.

Fig. 3 shows predicted time courses of arterial pressure (panel A) and of cerebral blood flow velocity (panel B), as derived by the stochastic (dashed lines) and deterministic (continuous line) model (1–8). These time courses are directly comparable (both in timing and in amplitudes) with non-invasive observation data for sitting-to-standing maneuvers reported in the literature (e.g. Fig. 2 of [20]). Predictions in Fig. 3 and Fig. 4 were in fact obtained by calibrating parameter values for a sitting-to-standing experiment: parameter values are reported in Table 1.

**Fig 5:**
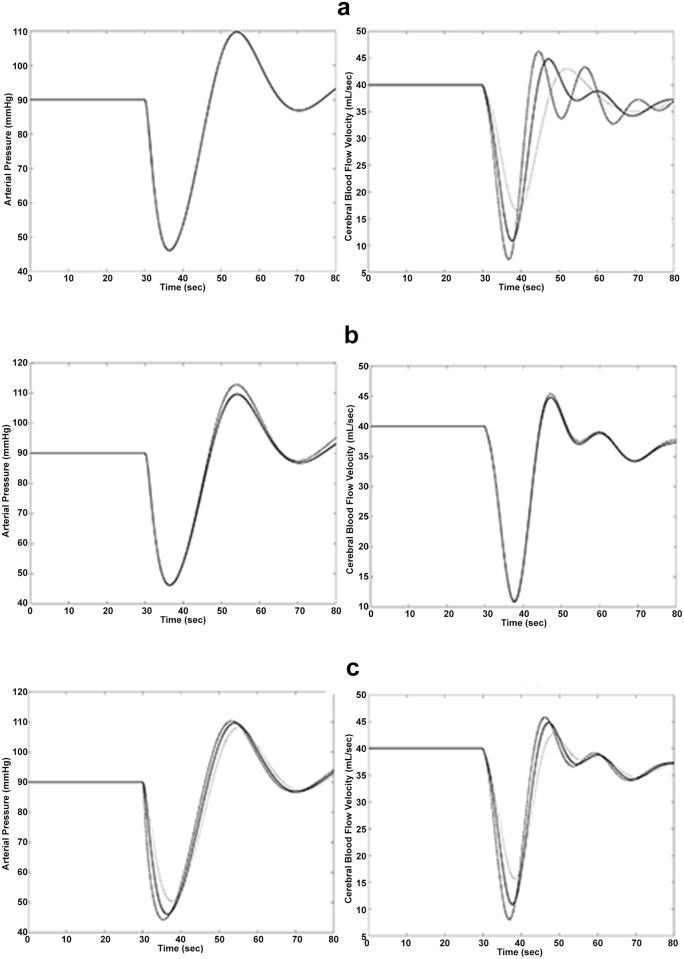
Simulated model behavior in abnormal physiological conditions obtained by varying some parameters. Panel A reports the Time course of model-simulated arterial pressure (mmHg), on the left, and the model-simulated cerebral blood flow velocity (CBFV, mL/sec), on the right, varying the parameter *k*_*xf*_. The continuous line describes the trend in the physiological situation (*k*_*xf*_ = 0.5), the dashed black line describes the trend with *k*_*xf*_ = 0.7 whereas the dashed gray line describes the trend with *k*_*xf*_ = 0.3. Panel B reports the time course of model-simulated arterial pressure (mmHg), on the left, and model-simulated cerebral blood flow velocity (CBFV, mL/sec), on the right, varying the parameter *k*_*h*_. The continuous line describes the trend in the physiological situations (*k*_*h*_ = 0.05), the dashed black line describes the trend with *k*_*h*_ = 0.07 and the dashed gray line describes the trend with *k*_*h*_ = 0.02. Panel C reports the time course of model-simulated arterial pressure (mmHg), on the left, and model-simulated cerebral blood flow velocity (CBFV, mL/sec), on the right, varying the parameter *k*_*xca*_. The continuous line describes the trend in the physiological situations (*k*_*xca*_ = 0.01), the dashed black line describes the trend with *k*_*xca*_ = 0.06 whereas the dashed gray line describes the trend for *k*_*xca*_ = 0.005.

In order to explore the behaviour of the model in medically meaningful situations, a series of simulations have been performed (Fig. 4, Fig. 5), making some parameters vary, one at the time, from normal to abnormal values. These simulations associate the variation of model parameters, as they could be physiologically postulated in relation to some dysfunction, with the time courses of pressures and blood flow velocity predicted by the model. Some of parameters affect more than others the behaviour of brain arterial pressure (BAP) or arterial pressure (AP) and cerebral blood flow velocity, as shown in Fig. 4 and Fig. 5.

For instance, Fig. 4 displays the time courses of BAPP and CBFV after sudden orthostatism, causing intravascular volume depletion, in correspondence of different values of the parameter *R*_0_ (equilibrium resistance before the maneuvre). Panel A shows that decreasing *R*_0,_ i.e. decreasing the equilibrium Peripheral Vascular Resistance, determines lower equilibria for brain arterial pressure. Panel B reports the time course of delayed brain arterial pressure for different values of *R*_0_ and shows that, while the pattern remains substantially unchanged, the lowest *R*_0_ value determines the curve at the bottom of the graph, which fails to rise up to the considered reference values. Panel C reports the trend of CBFV: despite lower values attained during the transient period for smaller values of *R*_0_, once the equilibrium is achieved the trend is not much altered, given the concurrent action of the other control mechanisms.

In panel D the greatest attainable delta in CBFV (the largest CBFV drop occurring in the transient phase), is reported as a function of *R*_0_, showing that cerebral blood flow velocity undergoes greater decrements in correspondence with smaller resistances.

Fig. 5 describes the behavior of the model corresponding to variations of the parameters *k*_*fx*_,*k*_*h*_ and *k*_*xca*_ in panels A, B and C respectively. The term $k_{xf}\tilde{F}(t)$ represents the local vasoconstriction, which is the fastest considered control mechanism: an increase in *k*_*fx*_ determines a faster decrease, but a more rapid increase (due to the introduction of the delay) in CBFV with respect to the standard situation. Further, the achievement of the equilibrium state (panel A) is slower (more oscillating). A decrease in the parameter, of course, produces a smaller and slower fall of CBVF along with a slower achievement of the physiological values, but a more rapid establishment of the equilibrium, with fewer and shallower oscillations.

The other two parameters represent the other two control mechanisms: on heart rate (*k*_*h*_) and on venous capacitance (*k*_*xca*_). Variations of these two parameters (that is variations of the efficiency of the sympathetic system and variations of central venous pressure recovery) influence very little both AP and CBFV (see panels B and C in Fig. 5).

## Discussion

Since cerebral autoregulation (CAR) indicates the ability of the circulation to adapt to variations in hydrostatic pressures in order to maintain constant and adequate perfusion to the brain, the study of the mechanisms and of the overall adequacy of cerebral autoregulation has meaningful implications for the assessment of the clinical severity of degenerative and cardiovascular diseases, such as the clinical states associated with chronic hemodynamic compromise (*e*.*g*. obstructive carotid artery disease). In these cases detection of impaired cerebral autoregulation might help to identify patients at risk, as already shown for cerebrovascular reserve capacity [4,30].

Assessing the adequacy of cerebral autoregulation is however not immediate. Empirical indices of CAR efficiency such as ARI, autoregulatory index and ARMA-ARI, autoregressive-moving average, [31] or indices derived from spectral analysis of the oscillatory pulse signal, for instance *G*_*r*_/ *G*_*c*_, the ratio of respiratory gain and the gain of the first cardiac harmonic [32], have been proposed in the past to offer semi-quantitative indications of a generically better or worse clinical situation. These indices have however a limited interpretability and their connection with well established physiological quantities is conceptually rather labile. Mathematical models of the response of the cerebral and systemic circulations to perturbation maneuvers could offer the opportunity to directly identify and quantitatively describe the key regulatory steps in the overall circulatory dynamics.

Mathematical models of the cardiovascular system and of cerebral autoregulation [10–19] have been developed over several years in order to describe the time courses of pressure and flow subsequent to postural changes. In general, many such models suffer from either one (or both) of the following shortcomings: they may be very large, consisting of many state variables bound to each other by interacting feedback loops; or, they may fail to fit available observations well. While the second shortcoming clearly determines major problems when foreseeing a possible clinical application of the model in question, the first shortcoming also gives rise to practical difficulties, connected with the difficult identifiability of overparameterized models from routine clinical data sets. In fact, the need exists, according to Panerai et al. [26], to develop multivariate models that take into account time-varying parameters with the aim to studying protocols to validate the reproducibility and ranges of the dynamic parameters of cerebral autoregulation.

The combination of the above considerations makes it so that it would be desirable to have a mathematical model, mechanistically representing accepted physiological phenomena, with as simple a structure as possible (but no simpler [33]) hence with as few free parameters to be estimated from data as possible, and still able to fit data well.

Attempting to respond to these requirements, the present work details a simple stochastic delay differential equations model of cardiovascular regulation during the postural perturbation maneuver (ESDeCAR-02). The model does in fact provide a realistic mathematical description of cardiovascular control, which may be fitted onto non-invasive experimental observations after postural changes, and is shown to replicate well observations performed during a sitting-to-standing manoeuvre.

The ESDeCAR-02 model attempts first of all to overcome the essential limitation of the first major model in this field, the Ursino-Lodi model [13], which considered the time course of arterial pressure as a forcing function, without explaining arterial pressure variations as a result of the hemodynamic changes induced by sudden orthostatism. In this way the new model uses the information content carried by the arterial pressure tracing in order to provide indications about the values of the autoregulation dynamics parameters.

While previous versions of pressure-flow-capacitance models [22] had significant problems explaining observations, the most recent Olufsen model [18] does an excellent job of reproducing observed arterial pressure and cerebral blood flow velocity tracings, and it is furthermore based on mechanical elements directly expressing relevant physiologic quantities (thereby making interpretation immediate). However, the Olufsen model is rather large and complex, making its use in routine clinical applications difficult.

The ESDeCAR-02 model is relatively simple, with only four differential equations (in place of 11) and only 7 free parameters to be estimated instead of the 62 of the Olufsen model.

An additional, relevant feature of the ESDeCAR-02 model is the stochastic modeling of pressure and flow variables, deriving from the representation of central venous pressure with a stochastic differential equation. This formalism is more appropriate to represent irregular variations of pressures and flows over time, produced by accidental muscle contractions, station adjustments, hormonal and neurological oscillations, respiration and swallowing, etc. The inclusion of system noise may be judged irrelevant (and hence the physiological system may be judged to be essentially deterministic), only after the volatility σ has been assessed, and it may very well be that for some subjects, but not for others, the inclusion of system noise could be indispensable to represent the evolution of the whole pressure-flow dynamics over time.

Finally, the model introduces explicitly distributed delays with respect to the four main controls (fastest to slowest: on local vasoconstriction, on heart rate, on peripheral vascular resistances, on venous capacitance), which may be presumed to depend on brain arterial perfusion pressure, consistently with generally accepted neurophysiological notions.

In the present work it has been shown that the ESDeCAR-02 model is well able to reproduce the time course of mean AP and CBFV after a sitting-to-standing change in posture, inclusive of some auto-correlated oscillations around the expected signals. In particular the model-simulated arterial pressure drops by approx 40 mmHg after standing, with a nadir at nearly 5–8 seconds post-maneuver, climbs back within another 10 to 15 seconds with an overshoot of approx 20 mmHg before stabilizing (Fig. 3, Panel A) and CBFV drops by approx 30 mL/sec after standing, with a nadir at 5 to 8 seconds post-maneuver, climbs back within another 10–15 seconds with an overshoot of approx 5 mL/sec before stabilizing (Fig. 3, Panel B). These features correspond point-by-point to what has been reported in the literature [20].

In order to explore the behaviour of the model in medically meaningful situations, a series of simulations has been produced, making single parameters vary in turn from normal to abnormal values (Fig. 4, Fig. 5). These simulations associate the variation of model parameters, such as could be physiologically postulated in relation to some dysfunction, with the time courses of pressures and flows which the model predicts would be observed in the presence of those dysfunctions.

All in all, the simulations appear very plausible: what is more important, they allow future validation or criticism of the model by comparison with experimental data sets, thereby paving the way to a future progressive refinement and calibration of CAR models.

While the present model’s structure appears reasonable, and its predictions seem consistent with observations, much work remains in fact to be done in assessing the possibility to use the model in order to discriminate between observation error and system volatility, and in determining the applicability of (simplifications of) this model to reliably estimate cerebral autoregulation in individual subjects under standard clinical conditions, which still remains an unsatisfied goal of biomathematical research in the neurosciences.

## Conclusion

The proposed empirical SDDE model of cardiovascular regulation after postural change, ESDeCAR-02, is able to reproduce observed average arterial pressure and cerebral blood flow velocity profiles, including autocorrelated random oscillations around the expected time courses. The model introduces explicitly distributed delays with respect to the four main controls (fastest to slowest: on local vasoconstriction, on heart rate, on peripheral vascular resistances, on venous capacitance), which may be presumed to depend on brain arterial perfusion pressure. Much work remains to be done in assessing the possibility to use the model in order to discriminate between observation error and system volatility, and in determining the applicability of (simplifications of) this model to reliably estimate cerebral autoregulation in individual subjects.
